# Immune Cell Functionality during Decidualization and Potential Clinical Application

**DOI:** 10.3390/life13051097

**Published:** 2023-04-27

**Authors:** Matthias B. Stope, Alexander Mustea, Nicole Sänger, Rebekka Einenkel

**Affiliations:** 1 Department of Gynecology and Gynecological Oncology, University Hospital Bonn, 53127 Bonn, Germany; 2 Department of Gynecological Endocrinology and Reproductive Medicine, University Hospital Bonn, 53127 Bonn, Germany

**Keywords:** decidua, fetal tolerance, implantation, placentation, early pregnancy development, NK cells, macrophages, innate lymphoid cells, reproductive immunology

## Abstract

Due to a vast influx in the secretory phase of the menstrual cycle, leukocytes represent 40–50% of the decidua at the time of implantation. Their importance for the implantation, maintenance of pregnancy, and parturition are known yet not fully understood. Thus, in idiopathic infertility, decidual immune-related factors are speculated to be the cause. In this review, the immune cell functions in the decidua were summarized, and clinical diagnostics, as well as interventions, were discussed. There is a rising number of commercially available diagnostic tools. However, the intervention options are still limited and/or poorly studied. In order for us to make big steps towards the proper use of reproductive immunology findings, we need to understand the mechanisms and especially support translational research.

## 1. Introduction

**Figure 1:**
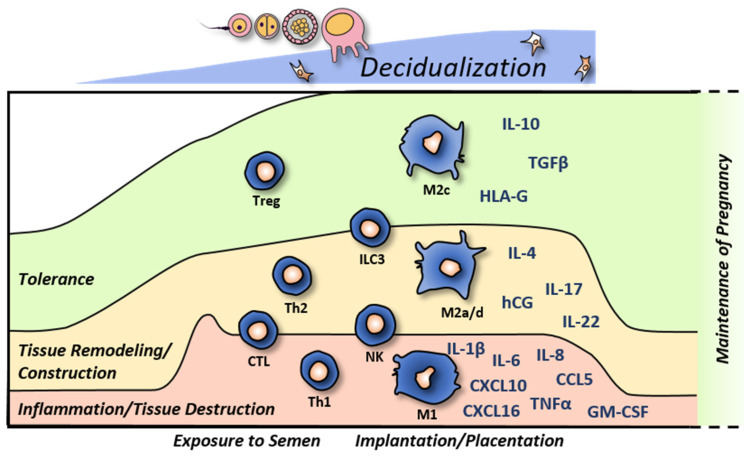
**Schematic immune changes during decidualization and early pregnancy.** During the menstrual cycle, the decidualization starts in the second half of the secretory. Exposure to semen and seminal plasma activates both inflammatory as well as anti-inflammatory mechanisms. Anti-inflammatory cells and factors (**green**) contribute to the tolerance towards semen and the fetus. This includes tolerogenic macrophages (M2c), regulatory T cells (Treg), interleukin (IL)-10, transforming growth factor (TGF)-β, and human leukocyte antigen (HLA)-G. Type 1 inflammatory processes (**red**) support mild tissue destruction, which is necessary for tissue remodeling during implantation and placentation. This includes T helper cells (Th)1, inflammatory macrophages (M1), and several factors, such as IL-1β, -6, -8, tumor necrosis factor (TNF)-α, granulocyte-macrophage colony-stimulating factor (GM-CSF) and the chemokines CCL5, CXCL10, -16. Together with rather type 2 inflammatory effects (**yellow**), tissue remodeling and angiogenesis are induced. This is mediated by “wound healing”-like macrophages (M2a/d), Th2, and factors such as IL-4, -17, -22, and human chorionic gonadotropin (hCG). In the decidual environment, otherwise, cytotoxic cells, such as natural killer (NK) cells and cytotoxic T lymphocytes (CTL), are less cytotoxic and rather secrete angiogenic factors. Moreover, cells such as innate lymphoid cells type 3 (ILC3) support both a microenvironment that favors tissue remodeling as well as mediating tolerance. All three immunological branches need to be tightly controlled in order to support the optimal development of early pregnancy. A shift towards tolerance or away from inflammation can cause shallow placentation and an insufficient remodeling of spiral arteries. A shift away from tolerance towards inflammation can cause the rejection of the conceptus. After completion of placentation, the immune balance shifts towards tolerance in order to maintain the pregnancy. Decidual tissue remodeling and, thus, inflammatory processes are barely needed anymore until the induction of labor.

For a successful pregnancy free of complications, maternal immune factors, including cells and soluble factors, must be precisely balanced [1]. On the one hand, the semi-allogeneic fetus expressing both maternal and paternal antigens must be tolerated. On the other hand, tissue remodeling is constantly accompanied by both destructive (type 1 inflammation) and constructive (type 2 inflammation) immune mechanisms (see Figure 1). Thus, implantation requires inflammatory actions, while protection against foreign antigens and pathogens must not be impaired at the fetomaternal interface [2].

Active tolerance mechanisms mediate the prevention of rejection of the sperm and, later, also of the conceptus. In contrast to passive tolerance due to a lack of immune mechanisms, active tolerance is involved in pregnancy [3]. The maternal immune milieu is characterized by immunomodulatory and anti-inflammatory factors, e.g., interleukin (IL)-4, IL-10, transforming growth factor (TGF)-β, leukemia inhibitory factor (LIF) and human chorionic gonadotropin (hCG). Their secretion occurs by the cells of the reproductive tract, embryonal cells, as well as by immune cells themselves. Interestingly, the reproductive, immune cells are adapted to their reproductive tasks and are, therefore, distinct from their peripheral pendants. They recognize foreign antigens but induce active tolerogenic responses and, thereby, induce the development of regulatory T helper cells and B cells [3,4,5].

Decidualization of the endometrium includes all processes that prepare the tissue for the implantation of blastocysts during the menstrual cycle [6]. The endometrium is composed of epithelial and stromal cells as well as secretory cells that form the glands. Furthermore, the endometrium is permeated by numerous vessels and has a strong blood supply. In relation to the ovarian cycle, decidualization begins after ovulation, when both hormones, progesterone and estradiol, increase. By that, specific processes are initiated [7]. Endometrial stroma cells differentiate into decidual stroma cells. In response to the hormones, decidual cells proliferate, and a receptive microenvironment is formed [6,8]. During decidualization, stromal cells secrete the Insulin-like growth factor binding protein (IGFBP)-1 and prolactin. These factors are also used as markers for decidualization in vitro. IGFBP-1 controls growth and development—especially under hypoxic conditions [9], as found in early pregnancy. Moreover, due to the decidual transition of the stroma cells, they secrete increasing amounts of IL-15 [10].

The process of decidualization and implantation parallels the initiation and progression of benign and malignant neoplasms. While cancer cells transform from epithelial to mesenchymal cells, the reverse takes place during decidualization. Endometrial fibroblastic stromal cells undergo mesenchymal-to-epithelial transformation [11], becoming epithelial-like cells. Similar to cancer cells, these exhibit high proliferative, anti-apoptotic capacities [12,13]. These processes are hormonally driven to varying extents [13,14,15]. Signaling cascades are also shared, including key regulators of cell growth (mitogen-activated protein (MAP) kinases, neurogenic locus notch homolog protein 1 (Notch-1), and Dickkopf-related protein 1 (Dkk1) [16,17,18,19,20]), cell motility (Notch 1 and homeobox protein A10 (HOXA10) [12,15,21,22]) and the interaction between the immune system. Similarly, to immune cells in the decidua, angiogenetic and invasive processes can be supported by immune cells in the tumor microenvironment. Both tumor and trophoblastic cells express immune inhibitory ligands, including B7 family molecules such as programmed cell death ligand (PD-L) 1, PD-L2, CD80, and CD86 [23,24], and T cell immunoglobulin and mucin-domain containing-3 ligand (TIM-3L) [25]. Similar to the immune cells, such as macrophages and NK cells, which support trophoblast invasion, the presence of tumor-associated macrophages (TAMs) is associated with tumor progression and metastasis [26,27]. In contrast, the presence of NK cells per se is no marker of tumor progression unless the phenotype is considered. Whereas cytotoxic NK cells show anti-tumoral effects, low-cytotoxic NK cells rather support tumor progress [25]. Moreover, higher prolactin levels, as found during decidualization, are also observed in several tumor types, especially in breast cancer [28].

The stromal cell reprogramming includes the downregulation of inflammatory capacity [29]. Moreover, immune cells are recruited to the decidualized tissue progressively, which participates in the functionalization of the decidua. On the one hand, immune cells are ultimately instrumental in implantation [6,30,31]. Natural killer (NK) cells and macrophages are found in close proximity to implanting trophoblast cells and support invading processes [32] accompanied by rather inflammatory factors. On the other hand, the semi-allogenic fetus must be tolerated. Several tolerance mechanisms take part in this process. The prerequisite for this is the general shift of the local immune micro milieu toward tolerance, which is accomplished by various factors. Decidual leukocytes are affected by seminal plasma if exposed to it, which contains several immunomodulating factors (e.g., TGF-β, soluble human leukocyte antigen (HLA)-G). Although foreign antigens of the semen lead to the activation of immune responses, these factors shift the leukocytes towards tolerogenic behavior. In humans, decidualization starts independently of conception in every menstrual cycle. After conception, decidualization continues and is additionally affected by the secreted factors of the embryo. Trophoblastic cells secrete TGF-β and especially hCG, which further affect the decidua and leukocytes. Moreover, in the first trimester before placental perfusion, vascularization and plugging of the maternal arteries prevents blood flow to the placental intervillous space. This creates and maintains a hypoxic environment [33]. Hypoxia also affects the decidual leukocytes by the stabilization of the transcription factor HIF (hypoxia-inducible factor). HIF regulates over 70 targets directly and, thereby, promotes angiogenic as well as tolerogenic milieu [34].

Due to the vast recruitment, the early decidua contains approximately 30–40% leukocytes, of which NK cells represent the largest subpopulation at 70%. The second most abundant leukocytic cell type is macrophages (20%) [35,36]. Other decidual immune cells include T cells, B cells, dendritic cells, and other innate lymphoid cells [35,37,38]. Immune cells support the processes of decidualization, implantation, and placentation.

The aim of this review is to provide an overview of the importance of the immune balance, which does not only include pro- and anti-inflammation but type 1 inflammation, type 2 inflammation (often named as anti-inflammatory because of its inhibitory actions on type 1 inflammation) and the tolerogenic mechanisms. It also provides a summary of the idea behind diagnostic efforts and their limitations, as well as the need for improvement concerning therapeutical options and especially the definition of suitable guidelines.

## 2. Decidual Innate Lymphoid Cells

**Figure 2:**
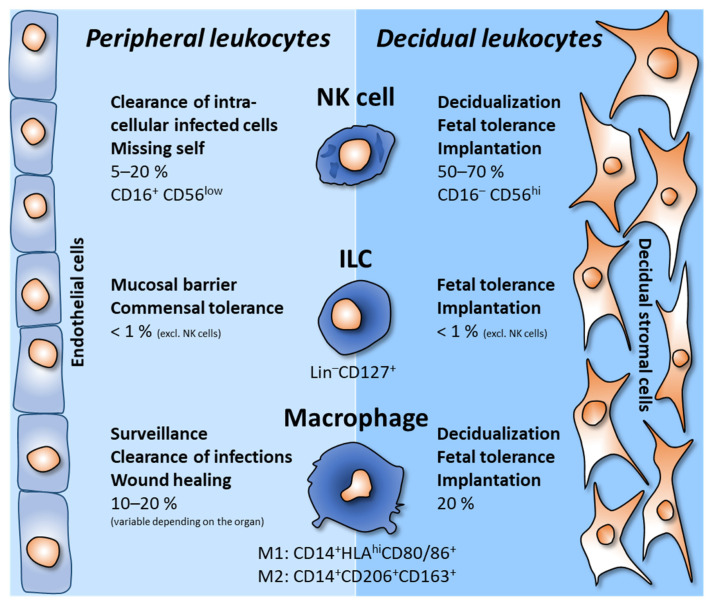
**Comparison of peripheral vs. decidual leukocyte tasks.** During decidualization, immune cells are recruited to the decidua and locally adjusted towards rather tolerogenic and implantation-supporting functions. Since they belong to the group of innate lymphoid cells (ILCs), NK cells are the most abundant cells in the decidua. The second most abundant cells are macrophages. Due to their vast secretory capacity, all the shown cells are not only adjusted by the decidual environment but also participate in the creation of an implantation-supporting milieu as well. Frequencies in the periphery and in the decidua in early pregnancy and phenotypic characteristics are shown.

The most abundant leukocytes in the decidua are NK cells (50–70%), which makes the uterus the organ with the highest frequency of NK cells in the body [33]. Interestingly, uterine NK cells differ vastly from blood NK cells. In the periphery, NK cells live up to their name as cytotoxic defenders—especially against infected and tumor cells. In contrast to peripheral blood NK cells, uterine NK cells do barely act cytotoxic but produce cytokines, growth factors, and chemokines during decidualization, receptivity, and implantation [38] (see Figure 2). Their metabolism and protein profile are unique when compared to peripheral NK cells [39]. Several factors contribute to the decidual phenotype of NK cells, including IL-15, TGF-β [40], and hypoxia [41].

Blood NK cells are activated through the Fc receptor CD16/FcγRIIIa to initiate antibody-dependent cell-mediated cytotoxicity (ADCC), which is not expressed in the uterine subset (CD16-CD56^hi^). Moreover, NK cells detect target cells with downregulated major histocompatibility complex (MHC)I escaping the recognition by cytotoxic T cells (referred to as “missing self”). Thereby, the cytotoxic behavior of NK cells is inhibited by MHCI. This mechanism plays a pivotal role in fertility. To maintain the decidual phenotype of NK cells, fetal extravillous cells express HLA-G and -E isoforms [42], which are less polymorphic, well-conserved, non-classical MHCI molecules. These pregnancy-related immunomodulators induce inhibitory decidual NK cell signaling and suppress the anti-fetal immune response [42,43]. In addition, HLA-G stimulates decidual NK cells to secrete various factors, such as vascular endothelial growth factor (VEGF), IL-6, and IL-8 [44,45]. These cytokines promote invasion and angiogenesis and are, therefore, central to decidualization and, eventually, placentation [46,47]. This makes NK cells essential in the remodeling of spiral arteries, which is necessary for proper placentation [48]. Moreover, decidual NK cells produce both pro- and anti-inflammatory cytokines, including interferon (IFN)-γ and tumor necrosis factor (TNF)-α as well as TGF-β and IL-10 [33,49], contributing to the local immune balance. Chemokines produced by decidual NK cells involving CCL5, CXCL10, and CXCL8 (IL-8) comply with several tasks. On the one hand, leukocytes are recruited to the decidua. On the other hand, NK cells guide the trophoblast during implantation in terms of the right direction and invasion depth into the decidua [50,51,52]. Therefore, NK cells are located in close proximity to trophoblast cells [32].

NK cells belong to the group of innate lymphoid cells (ILC). Recently, the involvement of the other ILC subtypes in processes at the fetomaternal interface (<1%; [53]) has been described [53,54]. ILCs correspond to T cells in terms of cytokine secretion but do not express T cell receptors (lin-CD127^+^ [55]; NK cells—cytotoxic T cells, ILC1-T helper (Th)1, ILC2-Th2, ILC3-Th17). However, the function and occurrence of the ILC 1–3 subtypes during pregnancy are largely unclear [53,56,57]. ILC3 has been the most studied and is the second most abundant ILC subtype in the decidua after NK cells. They produce the cytokines IL-8, IL-17, IL-22, and granulocyte-macrophage colony-stimulating factor (GM-CSF, CSF2), which also play important roles in pregnancy [58,59]. IL-8 and IL-17 support trophoblast invasion [60]. Low IL-22 and GM-CSF levels can be correlated with recurrent pregnancy loss [61,62,63,64]. Because ILC3 also promotes tolerance to intestinal commensals, similar tolerance-stimulating properties are also thought to occur during the stages of pregnancy. Although ILC3 can express MHCII, it is specifically downregulated in the uterus of mice and in the human decidua at term. TGF-β, hCG, and hypoxic conditions, which are major regulators in decidualization and early pregnancy, cause a downregulation of MHCII, which might contribute to fetal tolerance [55,65].

## 3. Decidual Macrophages

Since macrophages are key cells in tissue remodeling in both destructing and constructing processes, they are vital leukocytes due to the course of the menstrual cycle. In the decidua, macrophages form the second most abundant leukocyte population after NK cells (20%) [66]. Macrophages are phagocytes keeping homeostasis, can mediate antigen presentation, and participate in creating the immune milieu by cytokine production [67,68] (see Figure 2). During pregnancy, different macrophage subtypes accomplish the diverse tasks in the decidua [69,70]. However, the predominant differentiation stage of the decidual macrophages varies depending on the gestational age [71]. The beginning of gestation is more characterized by inflammatory mechanisms in the context of invasion and tissue rearrangement. At the time of implantation, macrophages resemble mainly pro-inflammatory M1 macrophages [72], which soon develop into tissue-remodeling M2a macrophages. During placentation, macrophages are located in the stroma near the invading trophoblasts and spiral arteries [32,73]. There, they support trophoblast invasion and spiral artery remodeling [74] by the secretion and regulation of the activity of matrix metalloproteinases (MMPs) [75]. These MMPs mediate the breakdown of the extracellular matrix, loosening the tissue integrity in order to rearrange it. Similar to decidual NK cells, HLA-G from extravillous trophoblasts induces macrophages to produce IL-6 and IL-8 in the first trimester [45]. By that, macrophages support angiogenesis and trophoblast invasion [76,77]. In addition, decidual macrophages secrete chemotactic molecules, cytokines, and growth factors to support placentation [78]. Furthermore, clearance of apoptotic degradation bodies by macrophages occurs [79,80,81,82].

After the implantation and placentation phase, immune cells mainly mediate fetal tolerance. This prevents fetal rejection. Trophoblastic cells secrete TGF-β, CXCL16, PD-L1, IL-10, and macrophage colony-stimulating factor (M-CSF, CSF1) for macrophage stimulation [81,82,83,84]. These factors differentiate the macrophages into an M2c-like phenotype. M2c macrophages secrete anti-inflammatory cytokines, including IL-10 and TGF-β, contributing to a tolerogenic milieu [71,85]. Due to the large number of macrophages at the fetomaternal interface but the low number of dendritic cells, macrophages are the predominant antigen-presenting cells at this site [86]. However, decidual macrophages have an altered antigen presentation potential and are more likely to mediate active tolerance [87].

The onset of contractions marks the end of pregnancy. Now the immunological milieu of the endometrium is modulated toward inflammatory processes [88]. Accordingly, macrophages differentiate back to the M1 phenotype and support inflammatory processes by secreting IL-6 and decreasing IL-10 and TGF-β levels [80,89].

## 4. Other Leukocytes in the Decidua

Dendritic cells (DC) represent appr. 2% of the decidual leukocytes [90]. Defense against infection is the main task for DC in non-pregnant women. Although present in a relatively low abundance, they interact with T cells, NK cells, and macrophages and thus regulate the more abundant cells at the fetomaternal interface. Thereby, DC supports the decidualization and implantation process [91]. Most significantly, during activation of T cells, DC determines the subtype, of which the balance is important for pregnancy success. DC is influenced by hormones, such as progesterone and estrogen, as well as hCG. hCG drives DC-mediated regulatory T cell (Treg) differentiation, whereas progesterone and estradiol rather support DC-mediated Th2 differentiation.

T lymphocytes are as well present at the fetomaternal interface [52]. In the CD4^+^ Th subset, Th1 lymphocytes can be detrimental to fetal tolerance when activated. In contrast, Tregs help to create a tolerogenic environment, and Th2 cells support the remodeling processes. The largest fraction of decidual T cells is CD8^+^ T lymphocytes [92]. These cytotoxic T cells have to be tightly controlled to not disturb fetal tolerance. Similar to their innate lymphoid cell pendants (NK cells), the decidual cytotoxic T cells differ from peripheral CD8^+^ T cells. They interact with inhibitory molecules such as HLA-C expressed by trophoblast cells and express significantly enhanced co-inhibitory molecules such as inhibitory killer cell immunoglobulin-like receptor (KIR), Tim-3 and cytotoxic T lymphocyte-associated protein 4 (CTLA-4) compared to peripheral CD8^+^ T lymphocytes supporting fetal tolerance [92,93].

Along with the other leukocytes, B cells are present in the decidua as well. They participate in the defense against infection but also support fetal tolerance. A subset of regulatory B cells (Bregs) secret anti-inflammatory cytokines supporting the tolerogenic niche. Moreover, B cells are able to express protective antibodies against paternal antigens to prevent rejection [94]. Antibody-producing B cells are also referred to as plasma cells.

Dendritic cells are also present in the human decidua an represent 1.7% of the leukocytes [90]. They show an immature phenotype [90,95]. In vitro, decidual dendritic cells mediate tolerance towards T cells [95] by secreting anti-inflammatory factors such as IL-10 under the influence of decidualized stromal cells [96].

## 5. Immune Implications in Adverse Pregnancy Outcomes

Inadequate decidualization can cause subfertility, infertility, and adverse pregnancy outcomes. The decidua creates a receptive environment which is needed for the attachment of the blastocyst, the invasion of trophoblast cells, and the placentation. Thereby, different immune types cooperate, including tolerance as well es tissue remodeling which integrates destructive (type 1 inflammation) and regenerative (type 2 inflammation) mechanisms, which have to be timely coordinated (see Figure 1).

Recurrent implantation failure (RIF) or recurrent pregnancy loss (RPL) can be caused by a disbalance towards type 1 inflammation due to either an increase in type 1 cytokines (i.e., TNFα or IFNγ), inflammatory cells (i.e. Th1) or cytotoxic activity by NK cells or a decrease in tolerogenic (i.e. Tregs, M2c macrophages, IL-10, TGF-β) or type 2 inflammatory mechanisms (i.e. Th2, M2a macrophages, IL-4, IL-13). Reasons for this imbalance can be manifold, including systemic immune alterations, genetic constitution, inflammatory effectors including immune disorders, stress, diet, physical exercises, and obesity, or altered activators including paternal factors, HLA matching, genetics of the embryo, and more. On a cellular basis, not only local immune cells but also trophoblast cells, as well as decidual epithelial and stromal cells, affect the balance by activating and inhibiting soluble and cell-to-cell-contact-mediated factors and receptors [24,42,51,52,97,98,99,100].

Detailed insight has already been provided by several reviews (i.e. in [101,102,103,104,105]).

## 6. Clinical Significance in Reproductive Medicine

The process of decidualization includes the proliferation and priming of endometrial stroma cells. This includes tissue remodeling and angiogenesis. The influx of leukocytes supports this structural adaption as well as the establishment of a receptive, tolerogenic milieu. General interventions to improve decidualization success are limited (see Table 1) but developing. The further sections aim to provide an overview of diagnostical and therapeutical tools targeting the immune balance during the decidualization process.

### 6.1. Targeting Regeneration

#### 6.1.1. Endometrial Scratching

Endometrial scratching causes a local injury that is aimed at activating the wound healing processes, which share mechanisms with decidualization [106]. However, the studies came to inconsistent conclusions. Several studies show an enhanced implantation rate after scratching [107,108,109,110,111,112,113,114,115,116,117,118,119,120,121,122,123,124,125,126,127,128,129,130,131,132,133,134,135,136,137,138], while there are also several studies that could not or barely show significant differences due to scratching [139,140,141,142,143,144,145,146,147,148,149,150,151,152,153,154,155,156,157,158,159,160,161,162,163,164]. Some of these studies even recorded a negative effect or considerable pain for the patient, clearly delivering arguments against endometrial scratching. As the enthusiasm conducting endometrial scratching is declining, the debate concerning the effect of endometrial scratching is still ongoing, where many factors play a potentially decisive role.

The timing of scratching throughout the cycle and the timing of the subsequent embryo transfer might be detrimental. Only a few studies addressed this in detail. A study showed better results when endometrial scratching was conducted in the luteal phase of the previous cycle compared to the follicular phase in the same cycle of the embryo transfer [110]. It was shown that endometrial scratching has a timely limited effect but is not restricted to the actual cycle. Until around 90 days after intervention, an improving effect was observed [111]. However, in a study where scratching in the proliferative (65.6%), periovulatory (69.6%), or secretory (64.3%) phases were compared, no significant differences due to the timing were seen [165]. Moreover, the form and force of the intervention might also affect the outcome. Peeling instead of scratching was shown to improve the pregnancy rate [112]. The aim of scratching is not to cause a vast injury but activate wound healing mechanisms due to a limited injury. Similar approaches are also used in other disciplines. In dermatology, microneedling is used to cause minimal physical trauma, which then activates regeneration due to the release of growth factors and stimulation of stem cells [166]. Whether similar mechanisms are also active in endometrial scratching remains to be elucidated. Nonetheless, molecular studies repeatedly showed an elevation of the expression of receptivity genes after scratching.

In several studies, endometrial scratching induced an elevation of pro- receptivity factors LIF [167], the homeobox proteins HOXA10 and HOXA11, as well as the cytokines IL-6, IL-8, IL-12, IL-13, IFN-𝛾 and monocyte chemotactic protein-(MCP-) 1 [116] as well as pro-angiogenic factors HIF, VEGF and the actual microvessel density [140].

These factors mediate and support receptivity and implantation. However, in patients with RIF LIF [168], HOXA-10 [169], HOXA-11 [170], HIF and the microvessel density [140] are significantly decreased. Restoration of these factors due to endometrial scratching might support implantation and placentation. On a molecular level, endometrial scratching builds a receptive microenvironment. The success of this intervention might, however, be dependent on additional factors. Scratching might support decidualization when there is a temporal or environmental reason, but not genetically or chronically altered decidualization. The identification of a suitable patient group could support the success of endometrial scratching.

For a more detailed insight, many reviews and meta-analytic publications were published concerning this topic, which seems to be declining but is still under a heated debate (reviewed i.a. in [171,172,173,174,175,176,177,178]).

#### 6.1.2. Platelet-Rich Plasma

The infusion with autologous platelet-rich plasma (PRP) is thought to support regeneration processes to improve thin endometrial lining found in patients with RIF [179]. PRP is found to be rich in growth factors, cytokines, and antibacterial peptides—especially after the activation of the platelets. This includes the tolerance-mediating TGF-β, pro-angiogenic VEGF, other growth factors such as platelet-derived growth factors PDGF, fibroblast growth factor FGF, insulin-like growth factor IGF1, IGF2 and epidermal growth factor EGF, inflammatory cytokines IL-8 and regenerative cytokines IL-4, IL-13 and IL-17 [180,181,182].

Thus, it locally facilitates regenerative mechanisms and benefits the whole decidualization process. In patients with thin endometrial lining, RIF, and recurrent miscarriage, the intrauterine infusion improves pregnancy rates and success effectively (reviewed i.a. in [183,184,185,186,187,188]). Comparing intrauterine infusion of hCG, G-CSF, PBMCs, or PRP, the infusion of PRP showed the most effective results in patients with RIF [189].

Activated PRP by thrombin and calcium chloride improves the in vitro behavior, including migration, invasion, and proliferation of endometrial cells [190]. Primary endometrial cells and cell lines show an increased expression of proteases, cytokines including IL-1α, IL-1β, and IL-15, and chemokines including CCL5, CCL7, and CXCL13 after PRP treatment [190]. These interleukins can activate an immune response. Since IL-15 is important for NK cell function, it supports the major decidual leukocyte subset. Proteases are necessary for tissue remodeling, which is essential in implantation. Chemokines recruit further leukocytes to the decidua to support its proper function. Moreover, it affects the hormonal levels supporting implantation success [191].

Although standardization of the method is still lacking, the intrauterine infusion with PRP is a promising tool supporting the regenerative capacity of the endometrial/decidual tissue.

### 6.2. Targeting Immune Balance

It is speculated that the majority of idiopathic infertility and (recurrent) pregnancy complications are caused by immunological disturbances. Genetic as well as environmental influences affect the immune cell’s ability to create the needed tolerogenic niche. There are diagnostic tools available. However, the therapeutical interventions, which directly target immune components, are still limited, or their application is not sufficiently tested [192,193].

#### 6.2.1. Diagnostic Tools

Several commercial tests are already available which directly or indirectly capture immune-related changes. Endometrial biopsies or pipelle samples can be tested for NK cell, Treg, and plasma cell counts [194,195]. Altered numbers in these immune cells can be an indication of an immune-related cause of infertility.

Elevated plasma cell (antibody-producing B lymphocytes) counts indicate chronic endometritis [196]. An altered endometrial microbiome or chronic infections can create a misregulated inflammatory environment, which impairs fertility. Commercial tests are available to sequence the microbial colonization of the endometrium. However, the treatment options are limited to antibiotics combined with pre- and probiotic support afterward [197]. This might help to establish a healthy microbiome in all body niches, including the uterus. It is thought that besides the occurrence of healthy or unhealthy species also, the quantity plays a critical role in the effects of the upper reproductive tract microbiome [198].

Not only the number but also the function of the immune cells affect the fertility. The activity of immune cells depends on external factors, involving soluble factors creating the local immune milieu and cell-to-cell contacts, as well as internal factors, such as the genetic variants and expression quantity of receptors. Classical MHCI molecules show a broad polymorphism, of which certain haplotypes were correlated with increased pregnancy complications. These can be addressed by the characterization of the HLA and KIR or its receptor KIR genotyping [199]. Certain HLA and their pregnancy-relevant receptor types are found to be associated with poor IVF outcomes, including disturbances in implantation, the formation of the placenta, or the maintenance of the pregnancy [200]. However, the significance is limited, and further research is necessary.

An altered immune milieu can also be caused by autoimmune responses referred to as autoimmune-related infertility. In this case, autoantibodies can be tested. This includes anti-cardiolipin antibodies, lupus anticoagulants, anti-β2-glykoprotein-I antibodies, anti-transglutaminase IgA, and anti-nuclear antibodies, which can be tested [199,201].

A ratio of TNFα^+^ or IFNγ^+^ and IL-10^+^ or IL-4^+^ T helper cells in the peripheral blood before treatment correlates with IVF success rate [202,203,204,205].

#### 6.2.2. Interventions

Besides the various causes for an enhanced inflammatory/rejecting uterine micromilieu, it can be treated with glucocorticoids, intravenous infusion of phospholipid-stabilized soybean oil (intralipid), anti-TNFα, immunoglobulins [206], tacrolimus or heparin.

##### Glucocorticoids

Glucocorticoids are steroid hormones which are implicated in various processes. Although the level of glucocorticoids released under stress can compromise fertility, the right dose and timing of glucocorticoid release promote relevant reproductive functions [207]. Glucocorticoids also show immune modulatory effects. In general, glucocorticoids are potent immunosuppressors. Specifically in the uterus, NK cells [208] and macrophages [209,210] express the glucocorticoid receptor. By exposure to glucocorticoids, uterine NK cells decrease in their number [211] and show lowered cytotoxicity [212]. Accordingly, prednisolone decreases NK cell cytotoxicity in vitro [213]. Prednisone also binds TNFα according to in silico analysis inhibiting the inflammatory action of TNFα [214]. These changes create a rather tolerogenic milieu preventing sperm or fetal rejection. Besides the immunological changes, dexamethasone increases the survival and the prolactin secretion [14] as well as the IFGBP-1 secretion [215] of primary endometrial stroma cells in vitro.

However, the success of peri-implantation glucocorticoid administration is still under debate [216]. Although the administration of prednisolone significantly decreases the pathologically elevated numbers of uterine NK cells in patients with RIF, it must not improve the pregnancy rates after treatment [217]. Due to the temporal changes in the immune requirements during decidualization and early pregnancy, the need for general immune suppression must be carefully considered. However, diagnostic tools revealing the immune milieu are limited and not standardly used [218]. More research is needed to develop appropriate guidelines for the administration of glucocorticoids in artificial reproductive techniques (reviewed i.a. in [219]).

##### Intralipid

Fatty acids show an immune suppressive effect. Thus, soybean oil, which is the active component of intralipid, causes an immune suppressive effect. The exact mechanism of this modulatory capacity is not clearly understood. It inhibits pro-inflammatory Th1 cells and the cytotoxic activity of NK cells [220,221]. In patients with RIF, the perfusion with intralipid decreased the endometrial immune activation [222], supporting a rather tolerogenic milieu. The success of intralipid has been summarized in several reviews (i.a., [223,224,225,226,227,228,229]). However, conflicting studies raise doubts on the effectiveness. In peripheral blood, a rather pro-inflammatory shift towards cytotoxic T cells was observed after intralipid treatment [230]. Other studies did not find an improvement in pregnancy and birth rates [231]. Further research is suggested in order to investigate the success of the administration of intralipid [225,232].

##### Tumor Necrosis Factor α Inhibition

TNFα is the major effector cytokine of the inflammatory TH1 immune responses. It shows a pleiotropic effect on various cell types and is especially involved in autoimmune responses. Immune as well as non-immune cells express the TNFα receptors and are affected by this cytokine [233]. Blocking or neutralizing antibodies (Adalimumab, Humira; Infliximab, Remicade; Certolizumab pegol, Cimzia; Golimumab, Simponi) or fusion proteins (Etanercept, Enbrel) against TNFα or its receptors prevent its inflammatory effector functions and can support a tolerogenic micromilieu [234].

In patients with RIF, etanercepts improve pregnancy and live birth rate [235].

Especially in subfertile women with elevated TNFα:IL-10 ratios concerning the T helper cells in peripheral blood [236] or increased peripheral NK cell numbers [237], anti-TNFα binding therapy decreased the inflammatory parameters and thereby increased the pregnancy and live birth rate.

##### Intravenous Immunoglobulin

The action of intravenous immunoglobulin is a result of a variety of mechanisms. Polyclonal immunoglobulin G (IgG) substitutes pathologic autoantibodies. It prevents the activation of antigen-presenting cells and shifts the T cell balance towards regulatory T helper cells. In sum, it downregulates the production of pro-inflammatory cytokines and supports a rather tolerogenic or balanced immune milieu [238,239,240]. Moreover, immunoglobulins suppress NK cell cytotoxicity in vitro [213].

The usage of IVIG in RIF and RPL can support fertility [241], especially in patients with known inflammatory pathologies, including NK cell changes in count or cytotoxicity [242,243,244,245] and Th1:Th2 ratio [240,246] (reviewed in [247,248,249]). In couples with recurrent IVF failure and HLA similarity, IVIG might also increase the chances of pregnancy [250], suggesting a rather immune-balancing than only tolerance-mediating effect of IVIG.

##### Tacrolimus

Tacrolimus is a calcineurin inhibitor, which is used to prevent organ rejection in transplant patients. Calcineurin inhibitors prevent the production of IL-2. IL-2 is a crucial autocrine signal in T cell development and proliferation. Thus, the treatment with tacrolimus prevents T cell-mediated inflammatory responses and increases anti-inflammatory cytokines [251]. Thus, in RIF patients with elevated Th1:Th2 ratio, tacrolimus improves the pregnancy and live birth rate [252].

##### Heparin

In addition to the beneficial effects on the decidualization of stromal cells, increasing the secretion of IGFBP-1 and prolactin [253], heparin also favors a regulatory T cell response [254], which might support the tolerogenic immune milieu. Heparin shifts the endometrial cytokines towards and implantation-supporting milieu by increasing the expression of IL-6 and G-CSF [255]. Moreover, heparin inhibits the activity of the inflammatory transcription factor NF-κB in endometrial stroma cells [256]. Thereby, heparin improves the live birth rate [257] in RIF [258] and RPL [259] patients and decrease in adverse pregnancy outcomes [260]. There are also reports which did not find any improvements due to heparin [261,262,263,264]. This could also indicate that the patients for whom this treatment is eligible or the intervention itself needs to be defined more precisely.

##### Granulocyte Colony Stimulating Factor

Granulocyte colony-stimulating factor (G-CSF; CSF3) is injected either subcutaneously or intrauterine. Locally it might improve endometrial receptivity, implantation processes, and angiogenesis. Thus, G-CSF can increase the live birth rate in patients undergoing IVF [265,266,267]. Although the exact mechanisms remain unclear, it is known that G-CSF is also produced during implantation. Moreover, in the decidua, the expression of its receptor increases pre-ovulatory. G-CSF signaling is involved in proliferation and differentiation and affects the Th2 cytokines and shifts the T helper cell balance towards regulatory responses. G-CSF is a strong inhibitor of cytotoxic NK cell function [268], which is necessary for the uterine receptive milieu. The success of G-CSF in increasing pregnancy rate has been reviewed in detail (i.a., in [267,269,270]). Although not all studies found an improving effect of intrauterine perfusion of G-CSF [271,272,273]. Thus, more research is necessary in order to define the working administration and patient group.

##### Intrauterine Injection of hCG

The intrauterine injection of hCG before intrauterine insemination (IUI) or embryo transfer (ET) can also shift the local balance towards a receptive, tolerogenic environment. However, several studies showed contradictory results [274]. It is speculated that this intervention only helps a certain group of patients which needs to be specified in further studies. The hCG priming of the leukocytes shifts their immune response to a rather implantation-supporting and tolerogenic phenotype. In patients with RIF, intrauterine administration of hCG increases the percentage of Tregs while improving the live birth rate [275]. Another option is to prime autologous peripheral blood mononuclear cells (PBMCs) with hCG ex vivo and re-inject them into the uterine cavity. This procedure increases the live birth rate in patients with RIF [276]. Despite the promising results, more research is needed [276,277,278].

##### Seminal Plasma and Paternal Antigens

Independently of fertilization and the fertile window, unprotected sexual intercourse shifts the local immune balance towards tolerance independently of the fertile window. The immunoregulatory components of seminal plasma affect the local cells [279,280].

There are more options to target the optimal balance between tolerance and immune activation against the paternal antigens. The induction of tolerance to paternal antigens is one factor which explains the correlation of the frequency of sexual intercourse with the conceiving rate. Similarly, the induction of mucosal tolerogenic immunity might also explain the increased conceiving rate by the exposure to semen by unprotected oral sex. As a therapeutic tool, active immunization with partner antigens became is suggested. Through active immunization with partner lymphocytes, the maternal immune system is aimed to get familiar and trained with the paternal antigens [281]. The immunological mechanisms are not clearly understood. It is speculated that the immune reaction against the paternal antigens is enough activating in terms of the production of anti-paternal cytotoxic antibodies (APCA). These antibodies, although with cytotoxic potential, are negatively correlated with recurrent spontaneous abortion (RSA) [282]. Their presence after the immunization might explain the elevated pregnancy rates. Probably, as no adjuvants are used, no too inflammatory responses are caused, which would induce rejection and harm fertility.

At the latest, with this example, it is striking that fertility is based on a fine balance between pro- and anti-inflammatory events.

Other conditions and interventions often indirectly affect systemic immune functions. For example, obesity creates a harmful systemic inflammatory milieu, whereas moderate physical exercise supports optimal systemic immune balance.

Besides these available options, there are barely coherently standardized recommendations regarding immunomodulatory therapies currently.

**Table 1:** Overview of immune-targeting interventions to support sufficient decidualization.

Diagnostics	Interventions	Objectives
NK cell count Plasma cell count Treg cell count Th1:Th2 ratios [194,195,202,203,204,205]	Glucocorticoids [219], intrauterine application of phospholipid-stabilized soybean oil [225,232], anti-TNFα [235,236,237], hCG infusion [276,277,278], immunoglobulins [247,248,249], tacrolimus [252], heparin [257,258,259], G-CSF [267,269,270] Immunization with partner antigens	Balanced tolerogenic Micromilieu Balanced inflammatory micromilieu
Microbiome [197]	Antibiotics, Pre- and Probiotics	Modify colonizers
Recurrent implantation failure Thin endometrial lining	Scratching [171,172,173,174,175,176,177,178], PRP infusion [183,184,185,186,187,188], G-CSF [267,269,270]	Wound-healing-like Decidualization, Regeneration

## 7. Summary and Outlook

The invasive implantation to build a hemochorial placenta in humans brings the fetal tissue in close contact with maternal tissue and immune cells. Instead of passive immunological ignorance, active tolerance is mediated by about 40–50% of the decidual cells, which are leukocytes. In order to avoid the rejection of the paternal antigens, these tolerance mechanisms actively create a tolerogenic niche. Decidual leukocytes, therefore, differ from their peripheral pendants.

Besides preventing rejection, decidual leukocytes support trophoblast invasion, tissue remodeling, and angiogenesis in order to build a sufficient placenta. These processes require locally and temporally limited inflammatory conditions. These are not comparable to the inflammatory conditions during inflammation which can cause vast destruction and, in the context of pregnancy, the rejection of the foreign structures, including the onset of labor resulting in abortions and pre-term labor. Thus, the decidual leukocytes must be optimal balanced to support pregnancy establishment, development, and maintenance (see Figure 2).

Although the immune components of the decidua and their relevance for pregnancy are known, translational routine implementations are lacking or are expandable. Further research is necessary to examine the actual pathologies, the effects of the interventions, and which diagnostics are necessary to find the suitable intervention for the individual patients. We suggest that attention to immunorelevant therapeutical interventions follow the rise of immunodiagnostics which are already available and find the recognition that it deserves in order to support the success of reproductive medicine.

## Data Availability

Not applicable.
